# Novelty-induced memory consolidation is accompanied by increased *Agap3* transcription: a cross-species study

**DOI:** 10.1186/s13041-023-01056-4

**Published:** 2023-09-25

**Authors:** Kristoffer Højgaard, Bianka Szöllősi, Kim Henningsen, Natsumi Minami, Nobuhiro Nakanishi, Erik Kaadt, Makoto Tamura, Richard G.M. Morris, Tomonori Takeuchi, Betina Elfving

**Affiliations:** 1 Translational Neuropsychiatry Unit, Department of Clinical medicine, Aarhus University, Aarhus N, DK8200 Denmark; 2 Danish Research Institute of Translational Neuroscience – DANDRITE, Nordic-EMBL Partnership for Molecular Medicine, Aarhus University, Aarhus C, DK8000 Denmark; 3 Neuroscience Research Unit, Mitsubishi Tanabe Pharma Corporation, Kanagawa, 227-0033 Japan; 4 Data Science Department, Mitsubishi Tanabe Pharma Corporation, Kanagawa, 227-0033 Japan; 5 NeuroDiscovery Lab, Mitsubishi Tanabe Pharma Holdings America Inc, Cambridge, MA 02139 USA; 6 Laboratory for Cognitive Neuroscience, Edinburgh Neuroscience, The University of Edinburgh, Edinburgh, EH8 9JZ UK; 7 Center for Proteins in Memory – PROMEMO, Department of Biomedicine, Danish National Research Foundation, Aarhus University, Aarhus C, DK8000 Denmark; 8 Gftd DeSci, Gftd DAO, Tokyo, 162-0044 Japan

**Keywords:** AGAP3, Novelty, Memory consolidation, Hippocampus, Dopamine, Dopamine receptor antagonist, Locus coeruleus, Immediate-early gene, Behavioural tagging, Synaptic tagging and capture hypothesis

## Abstract

Novelty-induced memory consolidation is a well-established phenomenon that depends on the activation of a locus coeruleus-hippocampal circuit. It is associated with the expression of activity-dependent genes that may mediate initial or cellular memory consolidation. Several genes have been identified to date, however, to fully understand the mechanisms of memory consolidation, additional candidates must be identified. In this cross-species study, we used a contextual novelty-exploration paradigm to identify changes in gene expression in the dorsal hippocampus of both mice and rats. We found that changes in gene expression following contextual novelty varied between the two species, with 9 genes being upregulated in mice and 3 genes in rats. Comparison across species revealed that ArfGAP with a GTPase domain, an ankyrin repeat and PH domain 3 (*Agap3*) was the only gene being upregulated in both, suggesting a potentially conserved role for *Agap3*. AGAP3 is known to regulate α-amino-3-hydroxy-5-methyl-4-isoxazolepropionic acid (AMPA)-type glutamate receptor trafficking in the synapse, which suggests that increased transcription of *Agap3* may be involved in maintaining functional plasticity. While we identified several genes affected by contextual novelty exploration, we were unable to fully reverse these changes using SCH 23390, a dopamine D_1_/D_5_ receptor antagonist. Further research on the role of AGAP3 in novelty-induced memory consolidation could lead to better understanding of this process and guide future research.

**Supplementary Information:**

The online version contains supplementary material available at 10.1186/s13041-023-01056-4.

## Highlights

Novel experiences affect gene expression in both mice and rats’ hippocampi.

Novelty-induced transcriptional changes affect widely differing genes in mice and rats.

Cross-species study finds *Agap3* mRNA upregulated in mice and rats’ hippocampi.

## Introduction

In our daily lives, we encounter numerous mundane things that are often forgotten within a few days [1]. However, such transient memories can be better retained if they are preceded or followed by novel and salient experiences [2, 3]. The beneficial effects of novelty on memory retention have practical implications as well, such as using novel experiences to improve learning and memory before regular lessons in school [4, 5]. These findings highlight the importance of understanding the molecular and cellular mechanisms underlying the role of novelty in memory in the brain.

Contextual novelty, referred to as exposure to a novel spatial environment, has been shown to increase transcription in the dorsal hippocampus first observed by Guzowski et al. [6]. It also enhances memory retention in hippocampus-dependent memory tasks [2, 7]. In experimental settings, exposure to a novel spatial environment shortly before or after the encoding of an independent memory can enhance the retention of a memory trace, such as converting transient 1-hr memories into long-term 24-hr memories [7, 8]. The beneficial effects of novelty are mediated by a neural circuit from the locus coeruleus (LC) to the dorsal hippocampus [9–11], leading to the activation of dopamine D_1_/D_5_ receptors and downstream signalling pathways in the dorsal hippocampus [9, 10, 12]. The increase in transcription in the dorsal hippocampus by contextual novelty [6, 13] may facilitate the *de novo* protein synthesis required for initial memory consolidation [7, 8, 14].

The molecular mechanisms underlying contextual novelty-induced memory consolidation can be explained by the synaptic tagging and capture (STC) hypothesis of protein synthesis-dependent long-term potentiation (LTP) [15–18]. During memory encoding, synapses that undergo potentiation are marked by a ‘synaptic tag’ by a post-translational process occurring locally at individual synapses [19]. An independent ‘event’, such as novelty, can activate a neuromodulatory circuit, subsequently leading to the production of plasticity-related products (PRPs) through the activation of dopamine D_1_/D_5_ receptors. If these receptors are activated in the same neuron shortly before or after the moment of memory encoding, the PRPs will be captured by the tagged synapses. This process then facilitates the consolidation of both structural and functional changes in the potentiated synapses, resulting in persistent LTP. The complete nature of PRPs has yet to be defined. While, newly synthesized protein in soma being captured by potentiated spines within the known time-frame of the STC, has been observed [20], a growing body of evidence suggests a crucial role for local translation in synapses [21, 22]. This implies that mRNA may be transported to and captured by the potentiated and tagged spine, thus both mRNAs and proteins might serve as PRPs [15, 23, 24].

Recent advancements in optical imaging techniques have enabled the investigation of structural and functional changes during LTP at a single synapse resolution [19, 25]. Structural plasticity is characterized by increased spine size, achieved through actin cytoskeleton rearrangement [26, 27]. This increase in spine size can be transient, decaying to baseline levels, or consolidate, a process thought to depend on a protein synthesis-dependent increase in the postsynaptic density (PSD) [26, 28–30]. Functional plasticity, on the other hand, is characterized by an increase in the number of AMPA-type glutamate receptors (AMPA receptors) in the PSD [31–33], mediated by the lateral diffusion of these receptors from extrasynaptic locations, followed by their subsequent ‘trapping’ within the PSD [34, 35]. The extrasynaptic pool of AMPA receptors is dynamically regulated by endo- and exocytosis. Maintainting an increased number of AMPA receptors in the PSD depends on enhanced exocytosis to recycle extrasynaptic AMPA receptors [36, 37]. Although several molecules, including Homer1a [20], AMPA receptors [38], brain-derived neurotrophic factor (BDNF) [39], activin A receptor type 1 C (ACVR1C) [40] and Atypical protein kinase C (PKMζ) [41] have been identified as PRP candidates (for a review, see Okuda et al. [17]), it remains unclear how the newly-synthesized PRPs mediate the consolidation of both structural and functional plasticity. Nonetheless, the STC hypothesis suggests that PRPs are captured by the synaptic tag in potentiated synapses, an interaction that stabilizes the structural and functional changes, ultimately leading to persistent LTP.

In the present study, our aim was to monitor transcriptional changes across a diverse set of genes following exploration of contextual novelty, with the objective of identifying potential, yet unexplored, PRP candidates. Given that memory enhancement induced by contextual novelty has been observed across multiple species, including mice and rats, we hypothesized that the critical genes involved would be common across these species. Therefore, we investigated contextual novelty-induced gene expression in the dorsal hippocampus in mice and rats. Furthermore, we analysed novelty-induced gene expression in the dorsal hippocampus of rats after administration of the dopamine D_1_/D_5_ receptor antagonist SCH 23390.

## Materials and methods

### An overview of experiment 1 and 2 is given in Fig. 1

**Fig. 1:**
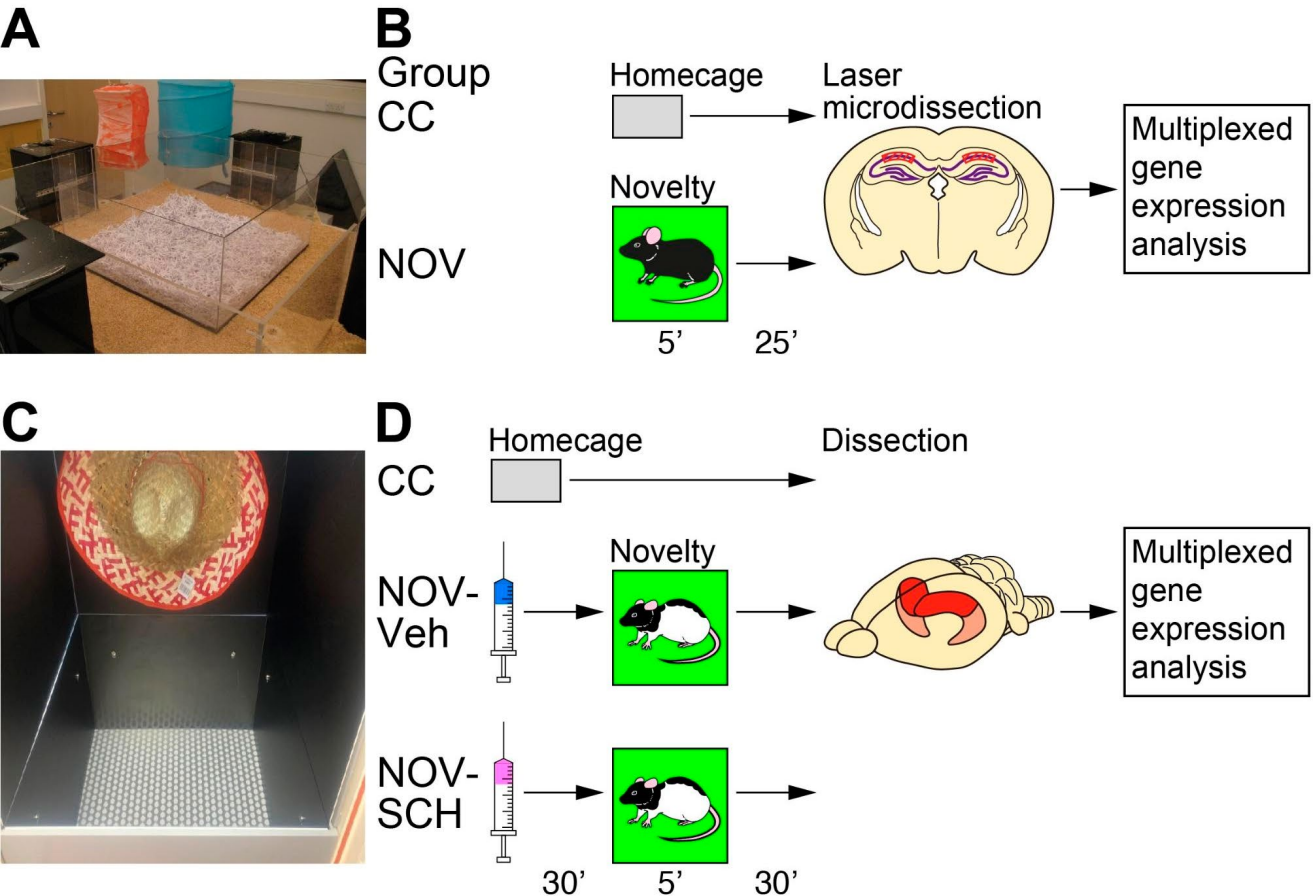
*Contextual novelty-induced gene expression in the dorsal hippocampus in mice and rats*. (**A**) Behavioral equipment for contextual novelty in mice. (**B**) Schematic overview of the mouse experimental design. Animals were divided into two groups in the critical session (Group CC, caged control; Group NOV, 5-min contextual novelty exploration; *n* = 5 in each group). Brain samples were collected 25 min after a 5-min novelty exploration, followed by laser microdissection of the hippocampal CA1 pyramidal cell layer and multiplexed gene expression analysis. (**C**) Behavioral equipment for contextual novelty in rats. (**D**) Schematic overview of rat experimental design. Animals were divided into three groups in the critical session (Group CC, caged control; Group NOV-Veh, 5-min contextual novelty exploration with Veh injection; Group NOV-SCH, 5-min novelty exploration with SCH 23390 injection; *n* = 10 in each group). Brain samples were collected 30 min after a 5-min novelty exploration, followed by dissection of the dorsal hippocampus and multiplexed gene expression analysis

**Experiment 1: Contextual novelty-induced gene expression at CA1 pyramidal cell layer in the dorsal hippocampus of mice**.

### Mice

We used 10 adult male C57BL/6 mice (Charles River, UK) aged 2 months. The mice were housed in pairs in standard cages with *ad libitum* access to standard laboratory chow and water. Housing conditions were maintained under a 12-h light/dark cycle (lights on 7:00 am), with no deviations from this schedule. All procedures were overseen by the University of Edinburgh Ethical Review Committee, compliant with the UK Animals (Scientific Procedures) Act 1986 (Project Licence P7AA53C3F) and with the European Communities Council Directive of 24 November 1986 (86/609/EEC) legislation governing the maintenance of laboratory animals and their use in scientific experiments.

### Exploration of novel environment in mice

For contextual novelty exploration, we used a square Plexiglas box (70 cm wide × 70 cm long × 30 cm high) that could be placed into the event arena for mice (120 cm wide × 120 cm long × 35 cm high) (Fig. 1A) [9]. Animals were handled for ~ 2 min per day for 2 consecutive days to familiarize them with the experimenter. For the 7 days leading up to the habituation period, all animals were housed in single-cages. Habituation to the box lined with sawdust as follows. In habituation session 1, pairs of mice were placed in the box together for 60 min; in sessions 2–4, pairs of mice were placed in the box together for 20 min each session; during sessions 5–7, individual animals explored the box for 5 min each session. Thereafter, for the critical test session 8, the mice were divided into 2 groups (*n* = 5/group): contextual novelty exploration group (Group NOV) and caged control group (Group CC). To introduce novelty, we changed the floor substrates from sawdust to cut-up straws, which was previously confirmed to enhance memory retention [9]. Group NOV was placed into the centre of the box lined with cut-up straws and allowed to explore freely for 5 min, after which they were returned to their home-cage for 25 min before their brains were collected. Group CC remained in their home-cage.

### Laser microdissection of the hippocampus

The mice were deeply anesthetized with sodium pentobarbital (1.4 ml per kg body weight by intraperitoneal injection) and perfused transcardially with 4% paraformaldehyde in 0.1 M phosphate buffered saline (pH 7.2). After excision from the skull, the brains were further immersed overnight in the same fixative. The fixed brains were dehydrated in ethanol and xylene in a vacuum infiltration processor (Tissue-Tek VIP E300; Sakura Finetek, Japan) and embedded in paraffin. The paraffin-embedded brains were cut into coronal sections (thickness: 10 μm) containing the hippocampal regions and mounted on polyethylene-napthalate membrane slides (Leica Microsystems, Germany). After deparaffinization, the sections were stained with 0.005% toluidine blue solution. Using a laser microdissection system (LMD7000, Leica microsystems), a pulsed UV laser beam was carefully directed along the borders of the hippocampal CA1 pyramidal cell layer, and the CA1 pyramidal cell layers were then collected from 40 sequential brain sections from each mouse.

### RNA purification and QuantiGene multiplex assay

Gene expression levels were determined using the QuantiGene 2.0 Multiplex Assay (Affymetrix, USA). Total RNA was isolated from laser microdissected samples using the QuantiGene Sample Processing Kit (Affymetrix) according to the manufacturer’s protocol. Total RNA was incubated at 54 °C for 18 h with working bead mix containing capture beads and 2.0 probe set. The next day, the beads and bound target RNA were washed and subsequently incubated with pre-amplifier at 50 °C for 1 h. After washing, the samples were incubated with the amplifier at 50 °C for 1 h. The samples were once again washed and incubated with the label probe at 50 °C for 1 h. Finally, the samples were washed and incubated with streptavidin-conjugated R-phycoerythrin at room temperature for 30 min. The resulting fluorescence signal was analyzed using a Bio-Plex Suspension Array System (Bio-Rad Laboratories, USA).

Seventy-six genes of interest were included based on the current understanding of LTP maintenance and our working hypothesis of PRPs (Additional file 1: Table S1). These genes include scaffolding and actin-binding proteins, guanine-nucleotide-exchange factors (GEFs), GTPase activating proteins (GAPs), kinases, and phosphatases among 1755 gene products that are enriched in postsynaptic dendritic spines [SynaptomeDB, http://metamoodics.org/SynaptomeDB/index.php [42]]. The immediate early gene (IEG) *Homer1a* was used as a positive control, while *Gapdh*, *Hprt*, and *Pgk1* were used as housekeeping genes. The data were normalized using the geometric mean of the expression of the three housekeeping genes.

**Experiment 2: Contextual novelty-induced dopamine D**_**1**_**/D**_**5**_**receptor-dependent gene expression in the dorsal hippocampus of rats**.

### Rats

A total of 123 male Long-Evans Th-Cre transgenic rats [43] backcrossed eight times to Lister Hooded strain were used as five experiment cohorts. The rats were split into groups of 6–20 and were housed in GM1800 DOUBLE-DECKER cages (Techniplast, Italy), with each cage containing 4 animals. The housing room was on a 12-h light/dark cycle (lights on 6 am), and the animals had *ad libitum* access to food and water. All procedures were approved by the Danish National Authorities (License number: 2018-15-0201-01405) in accordance with Danish and EU animal welfare legislations.

### Drugs

SCH 23390 hydrochloride (Cat. No. 0925; Tocris Bioscience, UK) was dissolved in sterile 0.9% saline (NaCl) to create a stock solution (2 mg/mL) and stored at − 20 °C. For the experiment SCH 23390 was diluted to 0.2 mg/mL. Vehicle (Veh) solution was sterile 0.9% saline. Animals received either a 0.2 mg/kg subcutaneous injection of SCH 23390 or similar volume of Veh.

### Apparatus

#### Object location memory task

An acrylic box (60 cm wide × 60 cm long × 50 cm high) with 3 black and 1 white walls was used for the object location task. The light intensity on the floor was adjusted to be between 100 and 110 lx. The arena was placed on a platform at the centre of the room with 3 spatial cues (lampshade, paper ball and plastic bell) hanging at various distances from the arena. The walls of the room were decorated with large and simple 2D shapes (black on white).

#### Novelty box

The novelty box is an acrylic box (60 cm wide × 60 cm long) placed in an enclosed environment (Fig. 1C). The box features black walls and is equipped with a novel floor substrate made of a metal grid. A large spatial cue is suspended above the novelty box. Light intensity within the box is adjusted to range between 20 and 40 lx.

### Object location memory task

The object location task used here is a modified version from Bayraktar et al. [44]. For the behavioral experiments 93 animals from 4 different cohorts were used. Handling started at week 4, to reduce anxiety and stress during the object location task. During weeks 4–7, the animals were transported to the behavioral room 2–3 times weekly and handled for 10 min per cage. At 8–9 weeks of age, they were habituated to various objects for 3 sessions using a large tub covered with sawdust containing 8–12 random objects (small toys of metal, wood or plastic). For the first object habituation session, animals were habituated to the acrylic box in groups of 4 and allowed to explore for 30 min. On sessions 2 and 3, they were habituated individually for 10 min.

The main experiment was conducted at 10 weeks of age. The animals were transported to the behavioral room and left quietly for 30 min. Animals were allowed to explore the arena without any objects for 10 min (Behavioral batch 1) or 20 min (Behavioral batch 2, 3 and 4) for 3 consecutive sessions. On session 4, they received a 20-min encoding trial in which 2 identical objects (empty brown beer bottles) were placed in 2 adjacent corners. After a retention period of either 1 or 24 h, one of the objects was moved to a novel corner and the animals were put back into the arena and allowed to explore for 5 min. All sessions were recorded on video and were scored live using automated tracking through TimeCSI (O’Hara & Co., Japan). Results were derived from the scoring of the first 2 min of both the encoding and the test trials.

### Exploration of novel environment in rats

A separate cohort of 30 animals was prepared for the gene expression experiment. From weeks 4 to 7, the animals underwent habituation, as described above. Starting at week 8, all animals were individually housed. During weeks 8–9, they were habituated to objects using the protocol described above. They were also habituated to the process of transportation and the termination room. Here, their cages were moved from the housing room to the termination room 2 times daily for 5 days. Each time, the cages were left in the termination room for 2–3 h for habituation purposes. The main experiment was conducted when the animals reached 10 weeks of age.

On the day of tissue collection, the 30 animals were randomly divided into three groups: Group NOV-Veh, Group NOV-SCH, and Group CC, each containing 10 animals. Group CC remained in their home-cages as per normal and were transferred to the termination room, where they rested quietly for 4 h before euthanization. Meanwhile, Groups NOV-Veh and NOV-SCH received subcutaneous injections of either Veh or SCH, respectively, 30 min before exploring the novelty box. Each animal was allowed to explore the novelty box for 5 min. Thirty min after this exploration, the animals were transported to the termination room and were immediately euthanized upon arrival by quick decapitation without anaesthesia. Their brains were removed, and the hippocampi were dissected and divided into dorsal and ventral halves before being rapidly frozen with crushed dry ice. Throughout this procedur, the time between picking up an animal and freezing its tissue did not exceeded 3 min.

### RNA extraction

Tissue was homogenized using the Precellys Evolution (6800 rpm, 3 × 30 s, pause 20 s; Bertin Technologies, France) and RNA was isolated using the miRNeasy mini kit (Qiagen, Germany) according to the manufacturers’ instructions. The quantity and purity of total RNA was measured using a NanoDrop-1000 spectrophotometer (Thermo Scientific, USA).

### Multiplexed gene expression analysis

A custom CodeSet of capture and reporter probes (NanoString Technologies, USA) was designed (full list in Additional file 1: Table S1). In addition, eight reference genes (*ActB, Ywhaz, Hprt, Aars, Mto1, Ccdc127, Tbp, and Rpl13a*) were included in the CodeSet. 50 ng of total RNA from each sample was hybridized to the capture and reporter probes for 20 h and then analyzed on the nCounter SPRINT platform (NanoString Technologies) according to the manufacturer’s instructions.

The raw data in CSV files were imported into nSOLVER 4.0 software (NanoString Technologies). ‘Systems Quality Control (QC)’ including imaging QC, binding density QC, and positive control linearity QC were performed on all samples using default settings. Then a ‘Positive Control Limit of Detection QC’ was performed using two standard deviations above the mean of the negative controls. No QC flags were detected from either the Systems QC or the Positive Control Limit of Detection QC. Raw data were then exported to Microsoft Excel (Microsoft Corporation, USA), and a background threshold was set (average of negative controls + 2 standard deviations). mRNAs with a mean value above the background threshold were selected for further analysis. The Normfinder algorithm was used on reference genes to select the 2 most stable genes (*ActB* and *Ywhaz*). The Normfinder algorithm is designed to rank gene expression stability across groups [45]. A positive control normalization was performed in nSOLVER, using the geometric mean of all positive controls except for the control named F, as per the manufacturer’s recommendation. Then, a second normalization was performed using the geometric mean of the Normfinder selected genes. The normalized data was exported to Excel for further analysis.

### Statistical analysis

All statistical analyses were carried out using IBM SPSS Statistics 28 (IBM, USA) and Microsoft Excel. Groups were tested for normality using the Shapiro-Wilk test and QQ plots. In case of violation of normality, the data was transformed for the analysis. A two-tailed Welch’s *t*-test was used to determine differences between groups. Due to performing multiple analysis and have a small sample size, quality control analysis were included to evaluate results. To control for false positives, false discovery rate (FDR) were calculated using the Benjamini-Hochberg method [46]. To investigate whether the statistical difference observed correlate to a meaningful difference, the standardized effect sizes were calculated and presented as Hedges’ *g* with 95% confidence interval (CI). Hedges’ *g* measures the difference between two group means and adjusts for small sample size bias.

## Results

**Experiment 1**.

### Contextual novelty-induced transcriptional changes in the mouse CA1 region of the dorsal hippocampus

Changes in mRNA expression for 76 genes of interest were investigated in the CA1 region of the dorsal hippocampus in mice following 5-min contextual novelty exploration (Fig. 1A, B). We have previously reported that this novelty context has a beneficial effect on memory retention in a manner dependent on hippocampal dopamine D_1_/D_5_ receptors [9]. In the multiplexed gene expression analysis, the Shapiro-Wilk test for normality and QQ-plot interpretation revealed that all groups exhibited a normal distribution. The expression of *Homer1a* mRNA was significantly higher in the group of mice that underwent a 5-min novelty exploration (Group NOV) (*n* = 5; 1.77-fold; Table 1) compared to the caged control group (Group CC) (*n* = 5), implying that our experimental setup was sufficient to detect changes in gene expression during novelty exploration. A key finding was statistically significant differences in mRNA expression between Groups NOV and CC for 10 of the 76 genes (Table 1 and Additional file 1: Table S2): Increased expression of 9 genes including *Arhgef2*, *Nsf*, *Lrrc7, Ppp1r9a*, *Dpysl3*, *Camk2d*, *Agap3 Ctnna1* and *Bai1*; decreased expression of *Rasgrf2*. According to the Benjamini-Hochberg FDR analysis, which serves as a safety measure when conducting multiple tests, no differences remained significant (Additional file 1: Table S2). However, the FDR analysis relies solely on the distribution of p-values, and since we have a small sample size, these p-values might not be entirely representative. Consequently, we used Hedges’ *g* to assess the magnitude of the differences in mRNA expression between Groups NOV and CC. Hedges’ *g* is commonly interpreted as having a large effect size when the value is greater than 0.8, as suggested by [47]. Taking into account the extremely large effect sizes observed for all significantly affected genes (Table 1), which imply meaningful changes, we decided to proceed with caution, being aware of the risk of false positives.

**Table 1:** *Novelty-induced transcription changing in the CA1 region of the dorsal hippocampus in mice.* Information on the fold change of mRNA expression relative to Group CC, the *p*-value from Welch’s *t*-test, and the results of a standardized effect size analysis, which includes Hedges’ *g* correction. CI, 95% confidence interval. The upregulated gene is highlighted in bold

Gene symbol	Gene expression	Welch’s *t*-test	Standardized effect size		
	Fold change	*p*-value	Hedges’ *g*	Lower CI	Upper CI
*Homer1a*	**1.77**	0.007	2.164	0.601	3.658
*Arhgef2*	**1.10**	0.008	2.002	0.491	3.444
*Nsf*	**1.21**	0.013	1.818	0.363	3.205
*Rasgrf2*	0.56	0.018	–1.777	–3.152	–0.335
*Lrrc7*	**1.13**	0.019	1.677	0.264	3.024
*Ppp1r9a*	**1.14**	0.021	1.682	0.267	3.030
*Dpysl3*	**1.12**	0.028	1.693	0.275	3.044
*Camk2d*	**1.41**	0.028	1.530	0.158	2.838
*Agap3*	**1.10**	0.031	1.512	0.145	2.815
*Ctnna1*	**1.22**	0.035	1.486	0.126	2.783
*Bai1*	**1.14**	0.048	1.334	0.013	2.594

**Experiment 2**.

### Dopamine D_1_/D_5_ receptor-dependent beneficial effect of post-encoding novelty on memory persistence in rats

The object location task is a behavioral paradigm used to detect hippocampus-dependent recognition memory of an object’s location within a familiar space, after one of the familiar objects has been moved to a novel location [48, 49]. This memory task relies on the animal’s noetic preference to seek out the object in the novel location if they remember it. Memory is measured by the preference for exploring the object that has been moved. Based on our previously published paper [44], behavioural batch 1 (n = 8/group) was trained using a weak encoding protocol (20-min encoding) for which memory decays over time to investigate the effects of post-encoding novelty exploration on memory retention. Rats were underwent a 20-min weak encoding trial, followed by a 40-min inter-trial interval. After this period, they were either allowed to explore a novel environment for 5 min (experimental group) or remained in their home-cages as usual (control group). In the 24-h memory test, no beneficial effect of contextual novelty on memory retention was observed (see Additional file 2: Fig. S1), suggesting that the strength of the memory was insufficient.

**Fig. 2:**
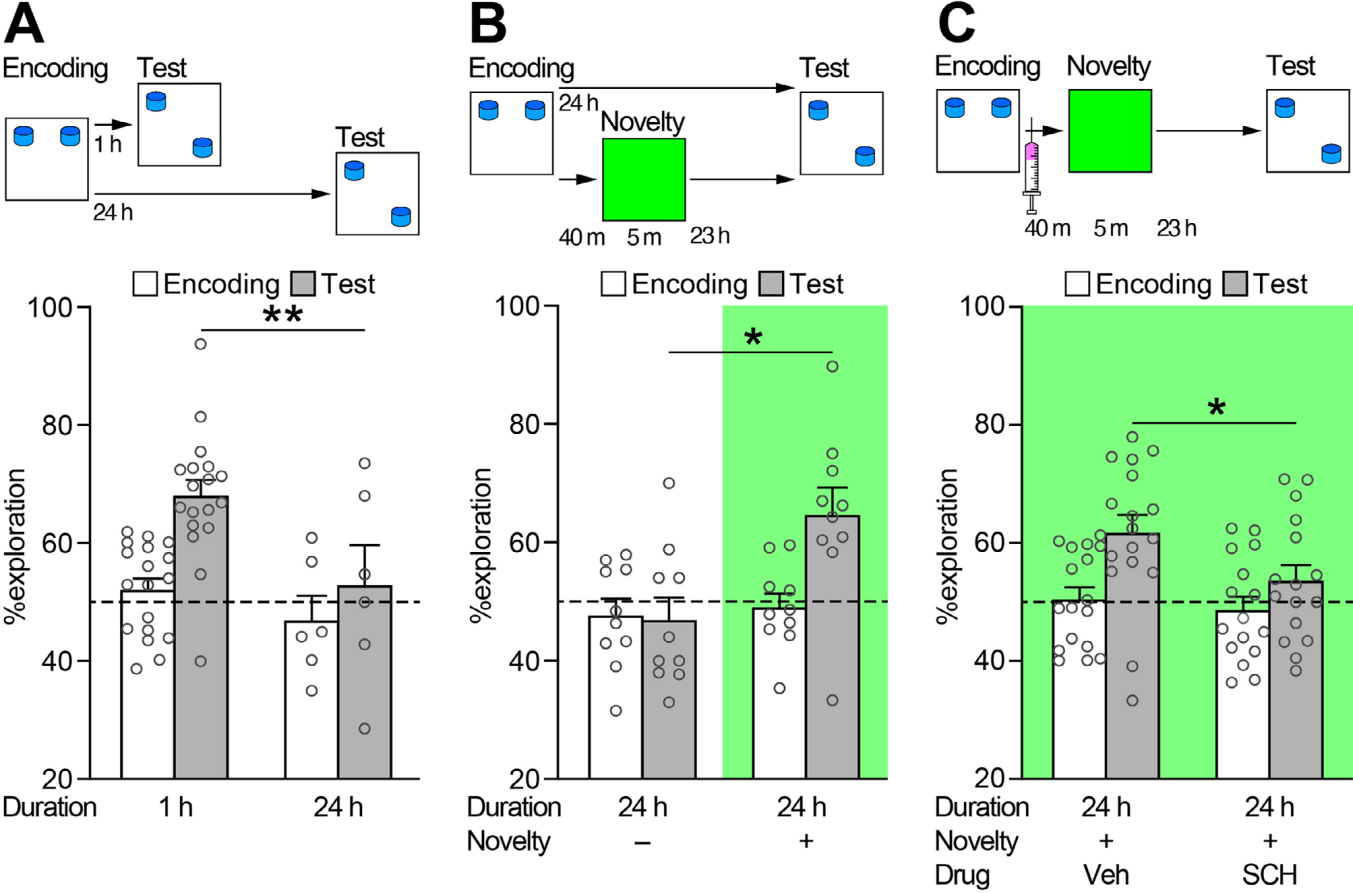
*Dopamine D*_*1*_*/D*_*5*_* receptor-dependent novelty-induced enhancement of persistence of object location memory in rats.* Graphs display the percentage of time spend exploring the object in the novel location, relative to the total object exploration time, during the first 2 min of both the encoding and the test trials. The dashed line represents the chance level. (**A**) A schematic of experimental design, along with the results from a 20-min encoding protocol, showing 1-h memory but not 24-h memory in behavioral batch 2. Welch’s *t*-test revealed a significant difference between 1-h (*n* = 18) and 24-h memory (*n* = 6) (68.10 ± 2.60% vs. 52.95 ± 6.73%; *t*_(22)_ = 2.576, *p* = 0.017). Effect size calculations demonstrated an extremely high Hedges’ *g*-value (*g* = − 1.172; lower 95% confidence interval (CI) = − 2.117, upper CI = − 0.205). Preference during encoding was unaffected by the experimental setup (52.16 ± 1.78% vs. 46.99 ± 3.71%; *t*_(22)_ = 1.160, *p* = 0.201). (**B**) A schematic of experimental design, along with the results showing the effects of contextual novelty exploration on 24-h memory in behavioral batch 3. Welch’s *t*-test indicated a significant increase in 24-h memory after contextual novelty exploration (*n* = 10) compared with controls (*n* = 10) (46.95 ± 3.71% vs. 64.73 ± 4.54%; *t*_(18)_ = 3.035, *p* = 0.007). Effect size calculations revealed an extremely high Hedges’ *g*-value (*g* = 1.300; lower CI = 0.346, upper CI = 2.225). Experimental conditions had no effects on encoding preference (49.08 ± 2.15% vs. 47.69 ± 2.62%; *t*_(18)_ = 0.389, *p* = 0.702). (**C**) A schematic of experimental design, together with the results showing the effects of dopamine D_1_/D_5_ receptor antagonist on contextual novelty-induced enhancement of memory retention in behavioral batch 4 is shown. Welch’s *t*-test revealed a significant decrease in 24-h memory for the SCH 23390-treated group (SCH, *n* = 16) compared with the vehicle-treated group (Veh, *n* = 17) (61.79 ± 2.95% vs. 53.67 ± 2.64%; *t*_(31)_ = 2.046, *p* = 0.049). Effect size calculations revealed a moderately high Hedges’ *g*-value (*g* = − 0.695; lower CI = − 1.378, upper CI = − 0.002). Experimental conditions did not affect preference during encoding (50.50 ± 1.90% vs. 48.70 ± 2.14%; *t*_(31)_ = 0.613, *p* = 0.546). All data are presented as mean ± SEM. *p < 0.05, **p < 0.01

To address this, behavioural batch 2 aimed to enhance contextual spatial memory by increasing the duration of each habituation session from 10 to 20 min. This change allowed the animals to become more familiar with the arena context and better able to recognize moved objects. It was confirmed that 20-min encoding resulted in a significant pattern of forgetting over a 24-h period (*n* = 6–18; Fig. 2A). Meanwhile, preference during encoding remained at chance for both conditions. Afterward, behavioral batch 3 was conducted to investigate the impact of contextual novelty exploration on memory retention in a 24-h memory test (*n* = 10 each). The study found that contextual novelty exploration significantly increased the animals’ preference for the object in the novel location, compared to the control group (Fig. 2B).

Lastly, behavioural batch 4 investigated the impact of blocking dopamine D_1_/D_5_ receptors during contextual novelty exploration on the enhancement of memory persistence typically induced by novelty. The 24-h memory protocol with contextual novelty exploration was used and either SCH 23390 or vehicle was administered systemically 30 min before contextual novelty exploration (*n* = 16–17 in each group). The systemic injection of SCH 23390 significantly reduced the beneficial effect of contextual novelty on 24-h memory retention (Fig. 2C).

### Contextual novelty-induced transcriptional changes in the rat dorsal hippocampus

After creating a contextual novelty condition suitable for inducing initial memory consolidation of object-place memory, we investigated the changes in gene expression in the dorsal hippocampus of rats induced by contextual novelty exploration (Fig. 1C, D). A multiplexed gene expression analysis was used to analyse the gene expression of an expanded list of 92 genes (see Additional file 1: Table S1) from Experiment 1, including *Arc*, *Fos*, and *Homer1a* as positive controls. With three groups of animals (Group CC, caged control; Group NOV-Veh, 5-min contextual novelty exploration with Veh injection; Group NOV-SCH, 5-min contextual novelty exploration with SCH 23390 injection), the study aimed to [i] investigate the effects of contextual novelty exploration on gene expression by comparing Group Nov-Veh with Group CC, and [ii] determine the contribution of dopamine D_1_/D_5_ receptors to contextual novelty-induced gene expression by comparing Groups NOV-Veh and NOV-SCH.

Based on the Shapiro-Wilk test for normality and QQ-plot interpretation, *Arc* and *Fos* mRNA expression violated normal distribution and were log-transformed for the analysis. All other groups showed a normal distribution. In the comparison between Groups Nov-Veh and CC, it was observed that the expression of *Arc*, *Fos*, and *Homer1a* mRNA was significantly higher in Group Nov-Veh (*n* = 10, Table 2) compared to Group CC (*n* = 10). This suggests that contextual novelty-induced changes in gene expression could be detected using our experimental setup. A principal result highlighted 10 genes with significantly altered expression: 3 upregulated genes, including *Ap3s2*, *Agap3* and *Dusp10*, and 7 downregulated genes, including *Tnik, Arfgef2, Cfl1, Rasgrf2, Wasf1, Capzb and Camk2a* (Table 2 and Additional file 1: Table S3). The Benjamini-Hochberg FDR analysis revealed that *Arc*, *Arhgef2*, *Fos*, and *Tnik* expression remained significant following the correction (Additional file 1: Table S3). However, standardized effect size analysis (Hedges’ *g*) uncovered a large effect size for all the significantly regulated genes (*g* > 0.9) (Table 2). Given the large effect size, all genes upregulated by contextual novelty exploration are considered relevant for further investigation.

**Table 2:** *Transcriptional changes in the dorsal hippocampus of rats following 5-min contextual novelty exploration.* Information on the fold change in Group NOV-Veh relative to Group CC, the *p*-value from Welch’s *t*-test, and the results of a standardized effect size analysis, which includes Hedges’ *g* correction. CI, 95% confidence interval. The upregulated genes are highlighted in bold

Gene symbol	Gene expression	Welch’s *t*-test	Standardized effect size		
	fold change	*p* value	Hedges’ *g*	Lower CI	Upper CI
*Tnik*	0.91	< 0.001	–2.272	–3.370	–1.137
*Arhgef2*	0.95	0.001	–1.695	–2.681	–0.670
*Arc/Arg3.1*	**1.85**	0.001	1.895	0.838	2.918
*Fos*	**3.97**	0.003	1.700	0.679	2.688
*Ap3s2*	**1.09**	0.007	1.352	0.391	2.285
*Cfl1*	0.93	0.012	–1.192	–2.103	–0.255
*Agap3*	**1.07**	0.013	1.211	0.271	2.124
*Rasgrf2*	0.91	0.016	–1.138	–2.041	–0.207
*Wasf1*	0.97	0.019	–1.104	–2.000	–0.179
*Capzb*	0.96	0.021	–1.089	–1.987	–0.165
*Homer1a*	**1.17**	0.022	1.131	0.201	2.033
*Camk2a*	0.95	0.023	–1.066	–1.961	–0.145
*Dusp10*	**1.11**	0.048	0.909	0.008	1.787

Since dopamine D_1_/D_5_ receptor antagonism inhibited contextual novelty-induced memory consolidation, we investigated the effect of SCH 23390 treatment during contextual novelty exploration on gene expression. A marginal decrease (< 10%) in the expression of 46 of the investigated genes was observed, as well as a moderate decrease (10–20%) in four genes (*Arc*, *Efnb*2, *Ezr* and *Fos*). However, when comparing gene expression between Groups NOV-Veh and NOV-SCH, no significant differences between groups were found (Additional file 1: Table S4).

### Cross species comparison

In this study, young mature animals—2-month-old mice and 10-week-old rats—were selected based on well-established patterns of brain development in these respective rodent species [50, 51]. Our hypothesis was that transcriptional changes induced by contextual novelty exploration would be conserved across these species, mirroring the observed similarities in behavioral outcomes. To validate this, gene expression data from individual experiments with mice and rats underwent a cross-species analysis. The first step involved listing genes with statistically significant *p*-values (*p* < 0.05) and large effect sizes (*g* > 0.8). Next, overlapping genes were identified, leading to the discovery of three shared genes: *Homer1a*, *Arhgef2* and *Agap3* (Table 3). *Homer1a*, a positive control, was expected to show significant changes in both species due to previous findings [52, 53]. Although *Arhgef2*’s transcription was affected in both species, its mRNA expression was downregulated in rats, suggesting it is unlikely to be a PRP candidate gene. Conversely, *Agap3* was significantly upregulated in both mice and rats. AGAP3, an ArfGAP, has been associated with AMPA receptor-trafficking and synaptic plasticity [54], making it a potential PRP candidate for regulating functional plasticity during initial memory consolidation.

**Table 3:** *Shared gene expression changes in mouse and rat.* A list of gene expressions affected by contextual novelty, shared between the mouse and rat dorsal hippocampus. Gene name, function, fold change, and statistical significance (Welch’s *t*-test) are included for both species. *p < 0.05; **p < 0.01. The upregulated genes are highlighted in bold

Gene symbol	Function	Mouse		Rat	
		CC vs. NOV		CC vs. NOV-Veh	
		Fold change	Significance	Fold change	Significance
*Homer1a*	Positive control, PSD scaffolding protein	**1.77**	**	**1.17**	*
*Arhgef2*	Rho/Rac GEF, Regulate actin assembly	**1.10**	**	0.95	**
*Agap3*	ARF6-GAP, Regulate AMPA receptor trafficking	**1.10**	*	**1.07**	*

## Discussion

The processes of memory encoding, storage and consolidation within the hippocampus have been extensively investigated, leading to significant progress in understanding the cellular and molecular mechanisms underlying memory. However, the transcriptional changes indispensable for initial memory consolidation have not yet been clearly defined. In this study, we conducted a cross-species comparison of contextual novelty-induced gene expression in the dorsal hippocampus. First, mice were exposed to a novel environment to investigate its impact on gene expression in the CA1 region of the dorsal hippocampus. We identified 10 genes with significantly altered expression, whose proteins are predominantly found in postsynaptic dendritic spines: 9 genes exhibited upregulated transcription levels, while 1 gene demonstrated downregulated expression. Second, we established a novel environment for rats suitable for enhancing spatial memory encoded during the object location paradigm [44]. Notably, the enhancement of novelty-induced spatial memory was inhibited by treatment with the dopamine D_1_/D_5_ receptor antagonist, SCH 23390. Third, when investigating novelty-induced changes in gene expression in the rat dorsal hippocampus, we identified 3 genes with upregulated transcription and 7 genes with downregulated transcription. None of these genes were significantly affected by the dopamine D_1_/D_5_ receptor antagonist. Fourth, when comparing the novelty-induced gene expression in the mouse and rat dorsal hippocampus, we found a substantially different set of regulated genes. Finally, our cross-species comparison revealed that the expression of 3 genes — *Homer1a*, *Agap3* and *Arhgef2* —was affected by contextual novelty in both mice and rats. However, it is important to note that *Arhgef2* was regulated differently in the two species. Although increased synthesis is a key criterion for PRP candidate [15, 17], this finding suggests that *Arhgef2* may play differential roles during initial memory consolidation, making it an interesting target for future research.

In 2007, Moncada and Viola pioneered the concept of behavioural tagging [7], presenting it as a behavioral analogue to the established the STC hypothesis. This framework allowed for a clear separation between two critical aspects of memory processing: [i] memory encoding and [ii] initial memory consolidation. They accomplished this by combining novelty exploration with a hippocampus-dependent object-location memory task. Research has demonstrated that such novelty-induced manipulations can induce gene expression [6, 53, 55]. Furthermore, studies have shown that inhibiting protein synthesis with anisomycin can compromise the initial memory consolidation [7, 8]. Based on these findings, it has been postulated that a ‘behavioral tag’ —similar to a ‘synaptic tag’ [16] —induced by weak memory encoding is essential for capturing PRPs [7, 14]. Notably, studies have reported that both behavioral tagging and STC mechanisms are governed by overlapping molecular pathways [8, 12]. This overlap emphasizes the value of investigating novelty-induced gene expression as a promising approach for identifying PRP candidates involved in initial memory consolidation. In accordance with these hypotheses, our research posits that exposure to novel experiences alone should be adequate for inducing the transcription of PRPs, independent of any synergistic relationship with memory encoding. In light of this, although our behavioural paradigm combines memory encoding and novelty exploration, we intentionally omitted encoding trials in our gene expression experiments for two main reasons: [i] our central aim is to identify PRPs whose gene expression is specifically triggered by novelty, and [ii] including encoding trials could introduce ambiguity, as there is existing evidence indicating that the encoding process itself can trigger gene expression [56].

It is important to carefully select and evaluate control groups when studying gene expression. For instance, Shires and Aggleton have questioned the suitability of using homecage conditions as a control when examining IEG expression in the hippocampus [57]. They found that swimming in a water maze led to higher *Fos* expression compared to homecage conditions. Moreover, it has been demonstrated that *Arc* expression increases in both novel and familiar environments [58]. However, our research is focused on PRPs, not IEGs. While much of the existing literature focuses on the expression of IEGs in familiar environments, studies specifically targeting non-IEGs like PRPs are notably scarce. Given our focus on PRPs, we consider the homecage setting to be a suitable control for evaluating PRP candidates. That said, it is important to exercise caution, as IEG expression is highly sensitive to external stimuli, even responding to something as minor as handling [59]. Thus, while homecage conditions may serve as an adequate control for PRP studies, the sensitivity of IEGs to environmental conditions warrants careful consideration. Adding complexity to this, Moncada and Viola demonstrated that CREB phosphorylation levels rise during exploration of a novel environment but decline in a familiar one [60]. These findings underscore the need for more nuanced control groups in future studies, particularly those focusing on PRPs.

Using contextual novelty exploration to induce gene expression, we identified *Homer1a* expression as being upregulated in both the mouse and rat hippocampus. This is in line with previous publications, showing novelty-induced *Homer1a* expression [52, 53]. Notably, Homer1a has been considered a PRP candidate, based on the landmark study by Okada and coworkers, who provided essential evidence supporting the STC hypothesis, where they showed activity-induced soma-derived Homer1a protein being transported to the potentiated spine [20]. However, subsequent research into Homer1a function has cast some doubt on classifying Homer1a as a PRP. Knock-out studies of Homer1a in mice have produced varied results, ranging from moderate effects on memory retention [61] to no observable effects at all [62]. In addition, Klugmann and colleagues compared the overexpression of all individual isoforms of Homer1 in the rat hippocampus [63]. They found that while Homer1g and Homer1c may have slight beneficial effects on learning and memory, overexpression of Homer1a had an inhibitory effect on learning and memory. Furthermore, memory deficits caused by Homer1a overexpression were accompanied by reduced LTP maintenance in the hippocampus [64]. Another line of studies suggests that Homer1a mediates homeostatic downscaling of surface AMPA receptors by regulating metabotropic glutamate receptor 1 (mGluR1) [65, 66]. Based on our current understanding, Homer1a would not be expected to be a PRP directly involved in the retention of both LTP and memory.

In addition to *Homer1a*, we identified *Agap3* as a potential PRP candidate. *Agap3* mRNA expression in the dorsal hippoampus was significantly upregulated in both the mouse and rat studies. AGAP3 is a multi-domain protein containing an N-terminal GTPase-like domain and a C-terminal ArfGAP domain, suggesting a bi-functional enzymatic activity. Multiple AGAP3 splice variants have been identified, including a full-size AGAP3 containing all functional domains and the smaller CRAG, a protein containing only the N-terminal GTPase-like domain [54, 67]. During neuronal development, the CRAG variant has been shown be crucial for axon guidance and protection from oxidative stress [67–70]. In the adult brain, both the AGAP3 and CRAG variants have been identified in PSD, forming a protein complex with the GluN2A subunit of the N-methyl-D-aspartate (NMDA)-type of glutamate receptor. The N-terminal GTPase-like domain is involved in activity-dependent AMPA receptor trafficking via SynGAP (Ras/Rap GTPase-activating protein) [54]. On the other hand, the C-terminal ArfGAP domain inhibits ADP-ribosylation factor 6 (ARF6) [54], a crucial regulator of endocytosis that recruits AP-2 and clathrin to the plasma membrane when activated [71]. Knockdown of AGAP3 led to an increase in ARF6 activity, resulting in an increase in the surface expression of AMPA receptors on cultured neurons in the rat hippocampus [54]. This is unexpected because previous studies have reported that ARF6, activated by ARF6-GEF, IQSEC2 (IQ motif and Sect. 7 domain ArfGEF 2), promotes the downregulation of the surface expression of AMPA receptors [72, 73]. The source of this discrepancy is unclear, but ARF6 activity is involved in multiple signalling pathways, and more research would be necessary for a full understanding [74]. Interestingly, inhibiting endocytosis of AMPA receptors has been shown to prolong the retention of both LTP and memory [75], similar to the beneficial effect of contextual novelty on the persistence of memories. We thus hypothesize that the recruitment of newly synthesized AGAP3 to the PSD of potentiated and tagged spines could promote the maintenance of LTP by inhibiting ARF6-induced endocytosis of AMPA receptors.

Numerous studies provide evidence that novelty-induced initial memory consolidation in the dorsal hippocampus relies on signal transduction mechanisms involving dopamine D_1_/D_5_ receptors, the activation of cAMP-dependent protein kinase (PKA), and extracellular signal-regulated kinases (ERKs). These processes ultimately initiate transcription, promoting the synthesis of essential proteins for the transformation of short-term memory into long-term memory in the behavioural tagging process [7, 8, 14, 76]. Crucial to this process is the detection of and response to contextual novelty, which have been associated with the activation of PKA and ERKs (p44 and p42 mitogen-activated protein kinases (MAPKs)), as well as the phosphorylation of the cAMP responsive element-binding protein (CREB) in the hippocampus [60, 77]. Dopamine D_1_/D_5_ receptors are intricately linked to this process in the CA1 region of the rat hippocampus, being coupled to PKA-p42 MAPK signalling and contributing to the regulation of phosphorylation of CREB, which may facilitate gene transcription [78]. The transcription of CREB-regulated IEGs, such as *Fos* and *Arc*, along with several other IEGs, has been shown to increase in the hippocampus when animals are exposed to a novel environment [6, 53, 55, 79, 80]. Importantly, obstruction of CREB function in the dorsal hippocampus inhibits long-term memory, while short-term memory remains unaffected in watermaze experiments [81, 82]. This has also been observed in contextual and trace fear conditioning [83]. Recent research further supports this, showing that engram-specific disruption of CREB function in the dentate gyrus of the dorsal hippocampus impairs consolidation of memory for contextual fear conditioning in mice [84]. In addition to transcription, somatic and/or local translation also play crucial roles in novelty-induced memory consolidation [7, 8, 14]. It has been demonstrated that the activation of dopamine D_1_/D_5_ receptors stimulates local translation in the dendrites of hippocampal neurons in vitro [85, 86]. This suggests the intriguing possibility that the dopamine D_1_/D_5_ receptor antagonist might influence memory consolidation in ways that extend beyond gene expression, such as involving somatic and/or local translation. This could explain why, in this study, SCH 23390 treatment only partially reversed novelty-induced gene expression while completely inhibiting the beneficial effect of novelty on memory persistence. While the evidence for local translation in the dendritic branches of hippocampal neurons is compelling [22, 87], our grasp of the physiological relevance of dopamine D_1_/D_5_ receptor-dependent local translation in contextual novelty-induced initial memory consolidation is still limited. Therefore, it is paramount to conduct further research to elucidate the role of hippocampal dopamine D_1_/D_5_ receptor-dependent local translation during novelty-induced initial memory consolidation, including the signalling pathway, temporal regulation, and the proteins involved.

An alternative explanation could be the lower statistical power derived from measuring gene expression across all cells within the area of interest, which might have resulted in a dilution of the observed effect. In the rat experiment, we collected samples from the entire dorsal region of the hippocampus, which contains various functionally distinct cellular subpopulations. Previous research has indicated that the CA1 region primarily receives novelty-induced dopaminergic signaling from the LC [88, 89]. In our sampling methodology, we collected multiple hippocampal areas, some of which might not be directly involved in the processes associated with the novelty signal. In the mouse experiment, we employed laser dissection to investigate gene expression specifically in the pyramidal cell layer of the CA1 region, focusing on a specific and relevant area. However, there are limitations using total RNA for detecting novelty-induced gene expression within the CA1 region, as it comprises multiple cell types, including interneurons and glia cells [90]. Moreover, memory encoding selectively involves a subpopulation of excitatory neurons in memory engrams [91]. Similarly, exposure to novelty has been shown to recruit neurons into engrams in a comparable manner, as indicated by the expression pattern of *Arc* RNA-positive neurons within the CA1 region [58]. Intriguingly, the memory-enhancing effects of novelty rely on the extent of overlapping populations between the novelty-engram and the engram encoding the memory being enhanced, and this effect is dopamine D_1_/D_5_ receptor-dependent [58]. When using lysate from hippocampal tissue, it is not possible to determine the number of *Agap3*-upregulated pyramidal neurons. Nevertheless, based on the literature [58], we expect novelty exploration to induce *Agap3* mRNA expression only in a subpopulation of pyramidal neurons within the CA1 region. Despite these limitations, the identification of upregulated *Agap3* mRNA expression in the dorsal hippocampus of both mice and rats using different protocols underscores AGAP3 as a potential PRP. To confirm the location and function of novelty-induced *Agap3* mRNA expression in initial memory consolidation, additional future studies will be necessary. Employing novel techniques in future experiments may facilitate understanding of engram-specific changes in transcription following contextual novelty [92, 93].

## Conclusions

Here, we propose a role for AGAP3 during contextual novelty-induced memory consolidation. AGAP3’s function fulfils the criteria of a PRP candidate. Additional studies will be necessary to confirm the exact contribution of AGAP3 during initial memory consolidation.

### Electronic supplementary material

Below is the link to the electronic supplementary material.

Supplementary Material 1

Supplementary Material 2

## Data Availability

The datasets generated and analyzed during the current study are available from the corresponding authors on reasonable request.
