# Supplementary figures and images for: Novelty-induced memory consolidation is accompanied by increased Agap3 transcription: a cross-species study
Source: Mol Brain. 2023 Sep 25;16:69. doi: 10.1186/s13041-023-01056-4 (PMC10521532; doi:10.1186/s13041-023-01056-4)

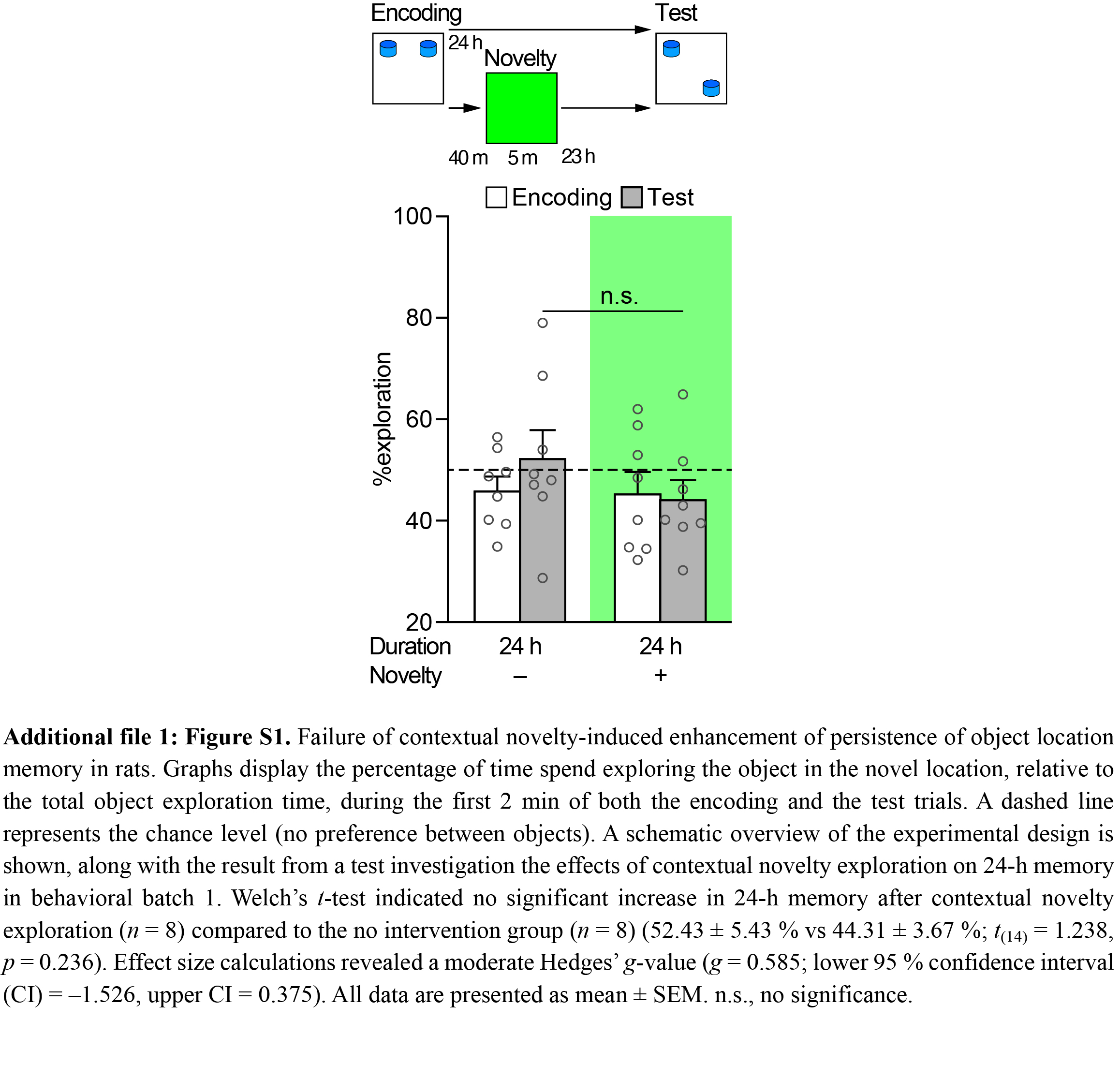

Supplement: Supplementary file 2 — Supplementary Material 2 [file 13041_2023_1056_MOESM2_ESM.jpg]
